# Dependency-like behaviors and pain coping styles in subjects with chronic migraine and medication overuse: results from a 1-year follow-up study

**DOI:** 10.1186/s12883-014-0181-4

**Published:** 2014-09-19

**Authors:** Bruno Biagianti, Licia Grazzi, Susanna Usai, Orsola Gambini

**Affiliations:** San Francisco Department of Veterans Affairs Medical Center, Building 16, 4150 Clement Street, San Francisco, CA 94121 USA; Department of Psychiatry, University of Milan Medical School and San Paolo Hospital, Milan, Italy; Headache Center, Neurological Institute C. Besta IRCCS Foundation, Milan, Italy

**Keywords:** Chronic migraine, Medication overuse, Pain locus of control, Dependency-like behaviors, Headache-related disability

## Abstract

**Background:**

Even after successful detoxification, 20-40% of subjects presenting chronic migraine with symptomatic medication overuse (CMwMO) relapse into medication overuse within one year. In this restrospective analysis on subjects referred to our center for detoxification, we investigated whether personality traits, dependency-like behaviors and pain coping styles predicted those who relapsed into medication overuse within the 12 months following the detoxification and those who did not.

**Methods:**

63 patients with CMwMO were assessed for personality traits, mood and anxiety, pain coping styles and dependency-like behaviors prior-to and one year after a detoxification program.

**Results:**

Of the 42 subjects who attended 1-year follow-up interviews, 11 relapsed into medication overuse despite a temporary benefit from detoxification and did not show clinical or psychological improvement, instead reporting increased anxiety and unmodified perpetuation of severe dependency-like behaviors. In contrast, subjects who did not relapse into medication overuse had clinical improvements that generalized to untreated domains, including decreased depressive symptoms and dependency-like behaviors, although showing unmodified low internal control over pain.

**Conclusions:**

Subjects who did not fall into medication overuse throughout the 12 months following the detoxification showed improved clinical, affective and dependence-related outcomes, but not pain coping strategies. Conversely, subjects who relapsed within one year into CMwMO continued to experience significant disability, pain intensity, and dependency-like behaviors. We believe that the persistence of maladaptive pain coping strategies and residual symptomatology increase the risk for recurrent relapses, against which pharmacological interventions are only partially effective. Further studies investigating predictors of relapse are needed to inform multi-disciplinary interventions for CMwMO.

## Background

Of the subjects with headaches referred to tertiary care clinics, as many as 15-30% are affected with chronic migraine with symptomatic medication overuse (CMwMO) [1]. Coping with the pain and disability associated with chronic migraine (CM) is likely to be a potential cause for increased medication intake [2,3]. However, some subjects with CM lose control over the use of symptomatic medications and develop counterproductive behaviors, including decision making deficits that compromise social functioning and negatively impact the quality of life [4-6]. The underlying pathophysiology of CMwMO is poorly understood. Evidence from recent studies suggests that neurobiological factors yet to be identified, including aberrant resting state functional connectivity within the default-mode network and reduced gray matter volumes of frontal regions, precuneus and hippocampus, could predispose certain subjects with CM to fall and recurrently relapse into CMwMO [7,8]. Currently, the initial line of treatment for CMwMO is detoxification from the offending drug [9]. Nonetheless, studies conducted in tertiary care clinics with patient populations and detoxification protocols similar to ours indicated that 20-40% of those subjects who discontinue medication overuse after a successful detoxification relapse into it within a period of months or years [10]. Longitudinal studies investigating variables associated with relapse following a successful detoxification have produced inconclusive result. Although the characteristics of CMwMO are considered to be outcome predictors associated with relapse [11,12], longitudinal studies showed that neither the frequency nor the intensity of migraines is significantly associated with relapse rates [10], instead suggesting that psychological and affective characteristics could play a key role. Mood disorders including depression have been considered relapse-predicting factors [13]: a 4-year follow-up study conducted by Hagen et al. found that lower scores for depressive symptoms at baseline were the only factor associated with functional improvement [14]. Furthemore, dysfunctional personality traits have been related to worsened long-term prognosis in subjects with CM^,^ and might facilitate the perpetuation of medication consumption [15,16]. Third, the severity of dependency-like behaviors could dampen the benefits of detoxification and increase risk to fall into CMwMO [17]. Finally, differences in attributing control over pain to particular influence the effectiveness of therapeutic interventions [18]. Thus, subjects with CMwMO who tend to attribute control over pain to internal sources are more likely to rationalize painful perceptions as less-frequent, less-intense, and less-distressing. Subjects who attribute pain control to external domains, such as fate, luck, or medical treatment, are conversely more likely to show maladaptive coping strategies leading to greater functional impairment [19,20]. We previously conducted an observational single-arm study on subjects with CMwMO referred to our headache center for a detoxification program. When looking at migraine characteristics and related disability prior-to, and then 1-year-after detoxification, we found that detoxification was associated with significant improvements in the group taken as a whole [21]. In this retrospective analysis, we decided to study comparatively those who did not fall into medication overuse throughout the 12 months following the detoxification and those who relapsed into CMwMO. In addition, we aimed to characterize personality traits, dependency-like behaviors and pain coping styles in the two subgroups and to determine whether these factors could be possible predictors of relapse into CMwMO.

## Methods

63 subjects with CMwMO were consecutively recruited at the Headache Center of the Foundation IRCCS Neurological Institute Carlo Besta in Milan. Subjects gave informed consent before participating and were informed of the absence of compensation. The study was approved by the IRCCS Neurological Institute Carlo Besta Ethics Committee. Inclusion criteria were: (i) diagnosis of chronic migraine, i.e. migraine headaches present on ≥ 15 days in a month on a regular basis for > 3 months, according to the current criteria reported in the International Classification of Headache Disorders, 2^nd^ Edition, and (ii) diagnosis of medicatio overuse, i.e. intake of simple analgesic on ≥ 15 days per month; or of any combination of ergotamine, triptans, analgesics, and/or opioids on ≥ 10 days per month on a regular basis for > 3 months [22]. Exclusion criteria were the following: other chronic pain conditions, pregnancy, progressive neurological disorders, present or past psychotic disorders, present or past substance and alcohol use disorders, and use of anti-psychotic drugs in the last 6 months. Prior to commencing the detoxification program, subjects underwent a battery of neuropsychiatric structured interviews and questionnaires. The detoxification program consisted of the following: (1) intravenous hydration for a period of 5 days; (2) intravenous steroids for the first 5 days followed by oral prescription for another 5 days; (3) diazepam orally in 2 prescriptions per day; and (4) ev- or im-metoclopramide or indomethacine when needed for intense rebound headache. On day 6, in consideration of their clinical features, medical history, and comorbidities, subjects could be prescribed with one or more prophylactic anti-migraine compounds recommended by published guidelines: flunarizine, pizotifen, propranolol, or amitriptyline [23]. At the conclusion of the detoxification program, subjects underwent a 3-hour psychoeducational training focusing on the correct management of NSAIDs and triptans during migraine episodes; additionally, they were encouraged to keep track of medications using a headache diary. 1 year after detoxification, the baseline assessment battery was repeated and subjects were categorized into two groups: Group 1 included those who did not fall into medication overuse throughout the 12 months following the detoxification; Group 2 included those who relapsed into CMwMO.

Preliminary findings from this sample regarding clinical improvements associated with detoxification were recently published [21]. For the purpose of this retrospective analysis, we focused on the following battery of assessments. The Migraine Disability Assessment Questionnaire (MIDAS) measures headache-related disability over the past 3 months by asking 5 questions about work, household- and social-related disability, the frequency of headaches, and the intensity of headache pain on a visual analog scale (VAS) [24]. The Structured Clinical Interviews for DSM-IV-TR (SCID) Axis I Mood Disorders and Axis II Personality Disorders assesses mood and personality disorders, 2002). The Hamilton Rating Scales for Anxiety and Depression (HAM-A, HAM-D) measure the intensity of depression and anxiety symptoms [25,26]. The Pain Locus of Control Scale (PLOC) measures the sense of perceived control over pain and health-related outcomes [18]. The PLOC Internal subscale measures the degree to which the individual perceives control over pain as internal, while the PLOC Chance and Powerful Others sub-scales are associated with strategies which attribute control over pain to either fate and luck or to health care providers. The Severity of Dependence Scale (SDS) identifies patterns of medication overuse and dependency-like behaviors among subjects with chronic headaches [17]. The Structured Clinical Interview for Substance Use Spectrum (SCI-SUBS) explores symptoms, behaviors, and experiences related to several domains, including substance use (including the use of rewarding substances), sensitivity to substances (including mood changes, anxiety attacks, and strange sensations), use of substances to alleviate mood or anxiety symptoms, tendencies toward strong emotions, and regulation of concentration at work [27].

For statistical analyses, we used the Statistical Package for the Social Sciences (SPSS, version 17.0; Chicago, IL, USA). We applied the Kolmogorov–Smirnov test to assess normality: clinical and psychopathological data were normally distributed. Due to the lack of significance in Levene’s test, continuous data were expressed by mean and standard deviation (M ± SD). We referred to levels of significance of *p*-value < 0.05. All tests were two-tailed. We used *t*-tests for paired samples separately in Groups 1 and 2 to identify any improvements in clinical and psychological measurements. We performed a binary logistic regression analysis to identify differences between Groups 1 and 2 at 1-year follow-up. In order to study risk factors for relapse into MO, we performed a series of binary logistic regression analyses for quantitative variables and a series of chi-squared tests for binary variables. Baseline variables used for these analyses included: gender, age, years with migraine, years with MO, frequency of headaches per month, number of tablets per month, intensity of headache pain, MIDAS total score, past history of psychiatric disorder, psychiatric diagnosis at time of evaluation, HAM-A and HAM-D scores, SDS score, and SCI-SUBS total score. In addition, we aimed to perform a logistic regression analysis based on the forward likelihood ratio method to estimate the strength of the associations between risk factors assessed at baseline and relapse into CMwMO at 1-year follow-up. Finally, we compared differences between pre- and-post-treatment PLOC scores using a *t*-test for paired samples, hypothesizing that detoxification would lead to increased perceptions of internal control over and decreased perceived external control.

## Results

Our sample comprised 63 subjects (81.0% female), with a mean age of 42.1 ± 10.2 years, and was characterized by a long history of migraine (years with migraine: 23.3 ± 13.1, range 1–60 years) and medication overuse (years with overuse: 2.8 ± 6.9, range 3 months-36 years). The intensity of headache pain, the frequency of headaches and medication intake were consistent extremely high rates of headache-related disability and severe dependency-like behaviors (SDS score: 8.7 ± 2.2; the cut off for SDS medication dependence is 5). See Table 1 for additional information. 21 subjects (38.7%) reported a past history of mood or anxiety disorder. 18 subjects (29%) were diagnosed with an anxiety or depressive disorder at baseline according to SCID-I criteria. We found no significant differences in clinical and psychological measurements between those who had been previously diagnosed with an anxiety or depressive disorder and those who had not. No subjects met the DSM-IV-TR criteria for personality disorders. The most-used symptomatic medications for migraine attacks were NSAIDs, triptans, or a variable combination of both. 20 subjects (31.7%) overused triptans, while 32 (50.8%) overused NSAIDs. The 9 remaining subjects (14.3%) who used both types of medication could not be further sub-classified. The analysis of variance according to the medication type did not show significant differences for clinical and psychological measurements. At baseline, 28 subjects (44.4%) were not receiving preventive treatment, and 35 subjects (55.6%) had previously been prescribed either single-compound or combination prophylaxis. After discharge, 31 subjects (49.2%) were newly prescribed with prophylactic medications. Within the one year following detoxification treatment, 31 subjects (49.2%, 25 females; age 41.1 ± 9.4) did not relapse into CMwMO and presented with an episodic migraine pattern (Group 1) [22], while 11 subjects (17.5%, 8 females; age 46.6 ± 10.1) relapsed into CMwMO despite a temporary cessation of overuse (Group 2). 21 subjects (33.3%) did not complete 1-year follow-up assessments (18 females; age: 41.2 ± 11.1). We were able to retrieve information about 17 of these subjects: 3 were refractory to treatment and required hospitalization, 4 fell into the exclusion criteria for either pregnancy (2) or a suicide attempt (2), and 10 did not to return to the clinic and declined to provide their clinical status by phone. The remaining 4 subjects did not attend their scheduled follow-up visit. A *t*-test for independent samples showed that there were no significant differences in measurements between subjects who completed the study and those who did attend their scheduled follow-up visit. In total, longitudinal analyses were carried out for 42 subjects (66.7%). Table 2A shows changes among clinical and psychological variables in Groups 1 and 2. One year following treatment, Group 1 subjects showed significant improvements in several domains, including frequency of headaches per month, number of tablets per month, MIDAS total score, intensity of headache pain, intensity of dependency-like behaviors and depressive symptoms. Conversely, Group 2 subjects did not show any clinical or psychological improvement and were significantly more anxious. Furthermore, dependency-like behaviors among Group 2 subjects were unmodified relative to baseline. Table 2B shows results of the logistic regression analysis: one year following treatment, subjects in Group 1 showed decreased tablet intake (p = 0.000), headache frequency (p = 0.000), headache-related disability (p = 0.024), anxiety (p = 0.024), depression (p = 0.027), and dependence-like behaviors (p = 0.000) compared to those in Group 2. To identify risk factors of relapse into CMwMO, a series of binary logistic regressions and chi-square tests was performed. However, none of the baseline clinical and psychological measurements were associated predicted relapse into CMwMO. Accordingly, no subsequent regression model could be built.

**Table 1:** **Baseline and 1-year follow-up characteristics for study completers and drop-outs**

	**Enrolled (N = 63) baseline**	**Dropouts (N = 21) baseline**	**COmpleters (N = 42) baseline**	**Completers (N = 42) 1 year follow-up**
Days with headaches per month	22.2 (6.5)	23.1 (6.9)	21.7 (6.4)	11.2 (7.4)
Tablets per month	33.0 (23.2)	38.8 (25.1)	30.1 (21.9)	15.0 (16.6)
MIDAS total score	68.9 (49.9)	79.0 (58.3)	68.8 (45.0)	38.6 (38.9)
Intensity of headache on VAS	7.7 (1.6)	8.1 (1.4)	7.6 (1.7)	6.5 (1.7)
HAM-A Score	12.8 (7.6)	13.3 (8.8)	12.6 (7.1)	11.6 (6.9)
HAM-D Score	12.4 (7.2)	12.4 (7.1)	12.4 (7.4)	9.8 (5.9)
SDS Score	8.6 (2.2)	8.8 (2.3)	8.6 (2.1)	5.7 (3.2)
SCI-SUBS total score	12.2 (9.1)	11.1 (7.5)	5.6 (3.3)	7.9 (7.9)

Values expressed as Mean (SD).

**Table 2:** **Clinical and psychological changes in Groups 1 and 2 (A) and between group differences at 1-year follow-up (B)**

	***Table A***						***Table B***
	**Group 1 (n = 31)**			**Group 2 (n = 11)**			
	**Baseline**	**1-year follow-up**	***t*** **-test** ***(p*** **)**	**Baseline**	**1-year follow-up**	***t*** **-test** ***(p*** **)**	**Logistic regression** ***(p*** **)**
Days with headaches per month	21.87 (6.3)	8.3 (5.5)	.000	21.4 (7.0)	19.3 (6.0)	.416	.000
Tablets per month	28.39 (19.7)	7.8 (4.4)	.000	34.7 (27.8)	35.5 (21.2)	.940	.000
MIDAS total score	63.6 (48.7)	30.3 (26.1)	.001	64.5 (34.5)	62.1 (57.8)	.907	.024
Intensity of headache pain on VAS	7.6 (1.8)	6.3 (1.7)	.001	7.3 (1.6)	7.1 (1.6)	.676	.216
HAM-A score	13.3 (7.6)	10.2 (5.9)	.014	10.5 (5.6)	15.5 (8.1)	.048	.024
HAM-D score	13.2 (7.9)	8.8 (5.5)	.001	10.1 (5.3)	12.6 (6.5)	.133	.037
SDS score	8.3 (2.2)	4.3 (2.4)	.000	9.3 (2.0)	9.4 (2.1)	.911	.000
SCI-SUBS total score	10.9 (7.2)	5.6 (3.3)	.000	14.6 (12.5)	7.9 (7.9)	.008	.191

Values expressed as Mean (SD).

Regarding the pain locus of control, the sample population (n = 63) exhibited an unbalanced PLOC profile at baseline with a significant predominance of the Powerful Others subscale over the Internal and Chance subscales. We found subjects’ migraine history to be significantly correlated with PLOC measurements as follows: the longer the history of migraine, the lower the internal control over pain (R = −0.326, *p* = 0.01), the lower the confidence in powerful others (R = −0.352, p = 0.01), and the higher the trust in chance (R = +0.307, *p* = 0.02). One year following treatment (see Table 3), study completers as a whole (n = 42) did not report increased internal control over pain, were still externally-oriented to powerful others, and showed a less-fatalistic approach to pain control. No significant differences between PLOC sub-scales were observed between Groups 1 and 2 either at baseline or at 1-year follow-up*.*

**Table 3:** **Changes in pain locus of control for study completers**

	**Pre-treatment (N = 42)**	**Post-treatment (N = 42)**	***t*** **-test**	
			**t**	***(p)***
PLOC internal	37.5 (10.7)	39.1 (11.1)	0.306 (−1.037)	
PLOC powerful others	48.1 (10.2)	46.7 (9.6)	0.451 (0.762)	
PLOC chance	35.5 (10.7)	29.2 (6.5)	0.000 (5.322)	

Values expressed as Mean (SD).

## Discussion

The recurrence of medication overuse that often accompanies the chronification of migraine is a critical factor in determining long-term functional impairment [28]. In this study, we first aimed to characterize the functional improvements resulting from a detoxification program among a highly-impaired sample of subjects presenting CMwMO and, second, to identify variables which can predict relapse into medication overuse following an effective detoxification treatment. Our sample was characterized by severe migraine features including: high frequency and intensity of migraine episodes, high medication intake, significant anxiety and depression levels, and dysfunctional dependency-like behaviors and pain coping skills.

The 1-year relapse rate for our sample was approximately in line with other studies [10-12]. Notably, within the group of subjects who discontinued medication overuse and presented at 1-year follow-up with episodic migraine, significant improvements generalized from migraine characteristics to untreated domains, including decreased depressive symptoms, headache related disability, and dependency-like behaviors [13]. In contrast, subjects who relapsed into CMwMO did not show clinical or psychological improvements, instead reporting increased anxiety and unmodified perpetuation of severe dependency-like behaviors. Furthermore, none of the baseline measurements predicted relapse into CMwMO.

We were particularly interested in assessing the PLOC among our sample. In line with previous studies on subjects with chronic pain [18], we found that, before the detoxification, the longer the history of CMwMO the lower are the perceived control over pain and the confidence in physicians’ pain management treatments. Increases in perceptions of internal pain control and decreases in perceptions of external pain control are both associated with successful completion of detoxification treatment and psychoeducation [19]. Unfortunately, our subjects showed unmodified low perceived control over pain and persisted in attributing it to powerful others and chance, irrespective of relapse into CMwMO. These findings suggest that detoxification and psychoeducation were not able to influence positively pain coping strategies among our sample.

Several limitations must be considered when interpreting the findings from this study. First, we acknowledge that the sample size at study completion did not allow sufficient power to detect effect sizes. Second, the high attrition rate can also account for the limited validity of these preliminary findings. Based on our prior experience recruiting subjects with CMwMO without any compensation [23], we approached our study design with a conservative estimate for drop-out rate of 20%. We believe that the severity of functional impairment associated with CMwMO and the intrinsic difficulty of conducting cohort studies without any compensation, people with a severe low-prevalence disorder are elements that strongly influenced attrition and sample size. Although we were inclined to believe that subjects who dropped out of the study had worse clinical and affective profiles, analysis of baseline differences between Groups 1 and 2 yielded no significant results.

In this retrospective analysis, we were not able to determine clinical and psychological predictors of relapse into CMwMO. Nonetheless, we believe that longer treatment trials in larger samples of patients with CM should be conducted to identify clusters of responders to detoxification protocols and to study biopsychological markers and neuroendophenotypes of recurrent relapses into medication overuse. In particular, emerging evidence supports the hypothesis that functional alterations of the default-mode network are associated with the perpetuation of counterproductive dependency-like behaviors, which are know to impair the clinical course of migraine [4,7].

Additional factors should be taken into consideration when identifying predictors of relapse within this patient population. First, the superimposition of medication overuse on CM complicates subjects’ clinical status to the extent that it might no longer be possible to segregate independent risk factors. The predictive value of the variables we collected may have been confounded by the reciprocal influences among the determinants of functional impairment. Furthermore, the lack of significant results could be attributed to the great variability among medication protocols targeting the complex residual clinical symptoms and psychiatric comorbidities among subjects with CMwMO. Although this variability complicates statistical pharmacological analyses, we believe that effective interventions for CMwMO must offer an individually-tailored and multi-disciplinary approach that targets the residual clinical symptoms or psychiatric comorbidities and incorporates both symptomatic and prophylactic polytherapies, and psychotherapeutic approaches [29,30].

## Conclusion

Despite the aforementioned limitations, findings of this exploratory study support the use of detoxification and psychoeducation to improve clinical, affective, and functional outcomes in a subgroup of subjects with CMwMO. However, these interventions appear to be ineffective in another subgroup of subjects, who continue to experience significant headache-related disability, pain intensity, and dependency-like behaviors. We believe that the persistence of maladaptive pain coping strategies and residual symptomatology increase the risk for recurrent relapses, against which pharmacological interventions are only partially effective. Further studies investigating predictors of relapse are needed to inform multi-disciplinary interventions for CMwMO.

## Abbreviations

CM: Chronic migraine
CMwMO: Chronic migraine with medication overuse
NSAID: Non-steroidal anti-inflammatory drugs
SCID: Structured Clinical Interview for DSM-IV
MIDAS: Migraine Disability Assessment Questionnaire
PLOC: Pain locus of control
SDS: Severity of dependence scale
SCI-SUBS: Structured clinical interview for substance use spectrum
